# Heterochrony and Cross-Species Intersensory Matching by Infant Vervet Monkeys

**DOI:** 10.1371/journal.pone.0004302

**Published:** 2009-01-28

**Authors:** Shahin Zangenehpour, Asif A. Ghazanfar, David J. Lewkowicz, Robert J. Zatorre

**Affiliations:** 1 Neuropsychology/Cognitive Neuroscience Unit, Montreal Neurological Institute, Montreal, Quebec, Canada; 2 Neuroscience Institute & Department of Psychology, Princeton University, Princeton, New Jersey, United States of America; 3 Department of Psychology, Florida Atlantic University, Boca Raton, Florida, United States of America; University of Sussex, United Kingdom

## Abstract

**Background:**

Understanding the evolutionary origins of a phenotype requires understanding the relationship between ontogenetic and phylogenetic processes. Human infants have been shown to undergo a process of perceptual narrowing during their first year of life, whereby their intersensory ability to match the faces and voices of another species declines as they get older. We investigated the evolutionary origins of this behavioral phenotype by examining whether or not this developmental process occurs in non-human primates as well.

**Methodology/Principal Findings:**

We tested the ability of infant vervet monkeys (*Cercopithecus aethiops*), ranging in age from 23 to 65 weeks, to match the faces and voices of another non-human primate species (the rhesus monkey, *Macaca mulatta*). Even though the vervets had no prior exposure to rhesus monkey faces and vocalizations, our findings show that infant vervets can, in fact, recognize the correspondence between rhesus monkey faces and voices (but indicate that they do so by looking at the non-matching face for a greater proportion of overall looking time), and can do so well beyond the age of perceptual narrowing in human infants. Our results further suggest that the pattern of matching by vervet monkeys is influenced by the emotional saliency of the Face+Voice combination. That is, although they looked at the non-matching screen for Face+Voice combinations, they switched to looking at the matching screen when the Voice was replaced with a complex tone of equal duration. Furthermore, an analysis of pupillary responses revealed that their pupils showed greater dilation when looking at the matching natural face/voice combination versus the face/tone combination.

**Conclusions/Significance:**

Because the infant vervets in the current study exhibited cross-species intersensory matching far later in development than do human infants, our findings suggest either that intersensory perceptual narrowing does not occur in Old World monkeys or that it occurs later in development. We argue that these findings reflect the faster rate of neural development in monkeys relative to humans and the resulting differential interaction of this factor with the effects of early experience.

## Introduction

To understand the evolutionary origins of a phenotype, we must understand the relationship between ontogenetic and phylogenetic processes [1], [2]. This relationship can inform questions about homology [3], [4] and help determine whether putative homologies reflect the operation of the same or different mechanisms [5]. For example, in primates, the ability to integrate the faces and voices of conspecifics during social interaction is critical to adaptive functioning. Indeed, the ability to perceive the intersensory invariance of facial and vocal expressions is present in adult and infant Old World monkeys [6], [7], [8], [9] and humans [10], [11], [12]. Although this apparent cross-species homology in the perception of intersensory invariance is interesting [13], it raises the following question: are the developmental processes leading to the emergence of these abilities similar or different across species [1], [3]? The most likely answer is that because of *heterochrony*—the fact that the rate of neural development in monkeys and humans differs—the developmental emergence of intersensory integration probably also differs across these two species.

There are at least three lines of evidence demonstrating that the rate of neural development in Old World monkeys is faster than in humans and that, as a result, they are neurologically precocial relative to human infants. First, in terms of overall brain size at birth, Old World monkeys are among the most precocial of all mammals [14], possessing ∼65% of their brain size at birth compared to only ∼25% for human infants [14], [15]. Second, fiber pathways in the developing monkey brain are more heavily myelinated than in the human brain at the same postnatal age [16] suggesting that postnatal myelination in the rhesus monkey brain is about three to four times faster than in the human brain [15], [16]. All sensorimotor tracts are heavily myelinated by 2 to 3 months after birth in rhesus monkeys, but not until 8 to 12 months after birth in human infants. Finally, at the behavioral level, the differential patterns of brain growth in the two species lead to differential timing in the emergence of species-specific motor, socio-emotional, and cognitive abilities [17], [18].

The heterochrony of neural and behavioral development across different primate species raises the possibility that the development of intersensory integration may be different in monkeys relative to humans. In particular, Turkewitz and Kenny [19] suggested that the neural limitations imposed by the relatively slow rate of neural development in human infants may actually be advantageous because the limitations may provide them with greater functional plasticity. This, in turn, may make human infants initially more sensitive to a broader range of sensory stimulation and to the relations among multisensory inputs. This theoretical observation has received empirical support from studies showing that infants go through a process of ‘perceptual narrowing’ in their processing of unisensory as well as multisensory information; that is, where initially they exhibit broad sensory tuning, they later exhibit narrower tuning. Specifically, young human infants can discriminate between different faces of another species [20] and between nonnative speech sounds [21], but this ability declines by the end of the first year of life. Likewise, 4–6 month-old human infants can match rhesus monkey faces and voices, but 8–10 month-old infants no longer do so and this narrowing persists into the second year of life [22]. Together, these findings indicate that as human infants acquire increasingly greater experience with conspecific human faces and vocalizations—but none with heterospecific faces and vocalizations—their sensory tuning narrows to match their early experience. It is interesting to note, however, that the ability to match monkey faces and voices seems to come back later in development in that adults can easily match monkey faces and voices and probably do so on the basis of simple temporal cues such as duration [23]. Although at first blush the adult findings might be seen as inconsistent with a perceptual narrowing account, it should be noted that, unlike adults, infants do not take advantage of duration cues and, as a result, fail to make such intersensory matches [22].

If a relatively immature state of neural development leaves a developing organism more ‘open’ to the effects of early sensory experience then it stands to reason that the more advanced state of neural development in monkeys might result in a different outcome. There are two possibilities. On the one hand, monkeys may be born with a perceptual system that is already tuned to a much narrower range of sensory input and, thus, may only be able to integrate the faces and vocalizations of their own species. This, in turn, would mean that they are ‘closed’ to the effects of early sensory experience and that they are ‘stuck’ with a narrowly tuned perceptual system. On the other hand, like humans, monkeys may be born with a perceptual system that is tuned to a broad range of sensory input but because of their advanced state of neural development may not be as open to the effects of early experience and, as a result, may be permanently tuned to a broader range of sensory input or the perceptual narrowing effect requires a greater amount of experience. In either scenario, monkeys would not be expected to exhibit perceptual narrowing effects in the same way, and at the same age, as humans do. No study to date has tested the theoretical possibility that, due to their precocial development, nonhuman primates may not exhibit the kind of narrowing of intersensory perception observed in human infants.

We tested this possibility empirically in the current study by investigating infant vervet monkeys' (*Cercopithecus aethiops*, an Old World monkey species) ability to match the faces and vocalizations of another species with which they had no prior experience. To make our results directly comparable to the human infant data [24], we used the same stimulus materials and employed the same testing procedures. Our vervet subjects ranged in age from 23 to 65 weeks, or ∼6 to 16 months, and were split into two groups (a younger group 23–38 weeks old, mean age = 33 weeks, or ∼8 months; and an older group 39–65 weeks old; mean age = 46 weeks, or ∼12 months). This was done to have overlap with the age range used in the human infant study [24]. The mean age of our subjects was 40 weeks (or 10 months) which, neurologically, would be the equivalent of ∼120 to 160 week-old humans [16].

Three mutually exclusive outcomes are possible in this study. The first is that all vervet infants (regardless of age) fail to exhibit cross-species face-voice integration. This would suggest that, perhaps because of prenatal experience with conspecific sounds, they were born with a narrowly tuned perceptual system. A second possible outcome is that the younger but not older vervet infants exhibit cross-species intersensory matching. This would indicate that the developmental timing of perceptual narrowing in vervets is similar to that found in human infants and that the influence of experience is powerful enough to override the reduced plasticity of the precocial brains of monkeys. The third and final possible outcome is that perceptual narrowing does not occur in vervets and, as a consequence, both younger and older monkeys exhibit cross-species intersensory matching. This final outcome would indicate that vervets' intersensory sensitivity remains broadly tuned in spite of their postnatal experience and would suggest that vervets' precocial brains are less sensitive to the effects of early social experience than the brains of their human counterparts.

## Results

**Figure 1:**
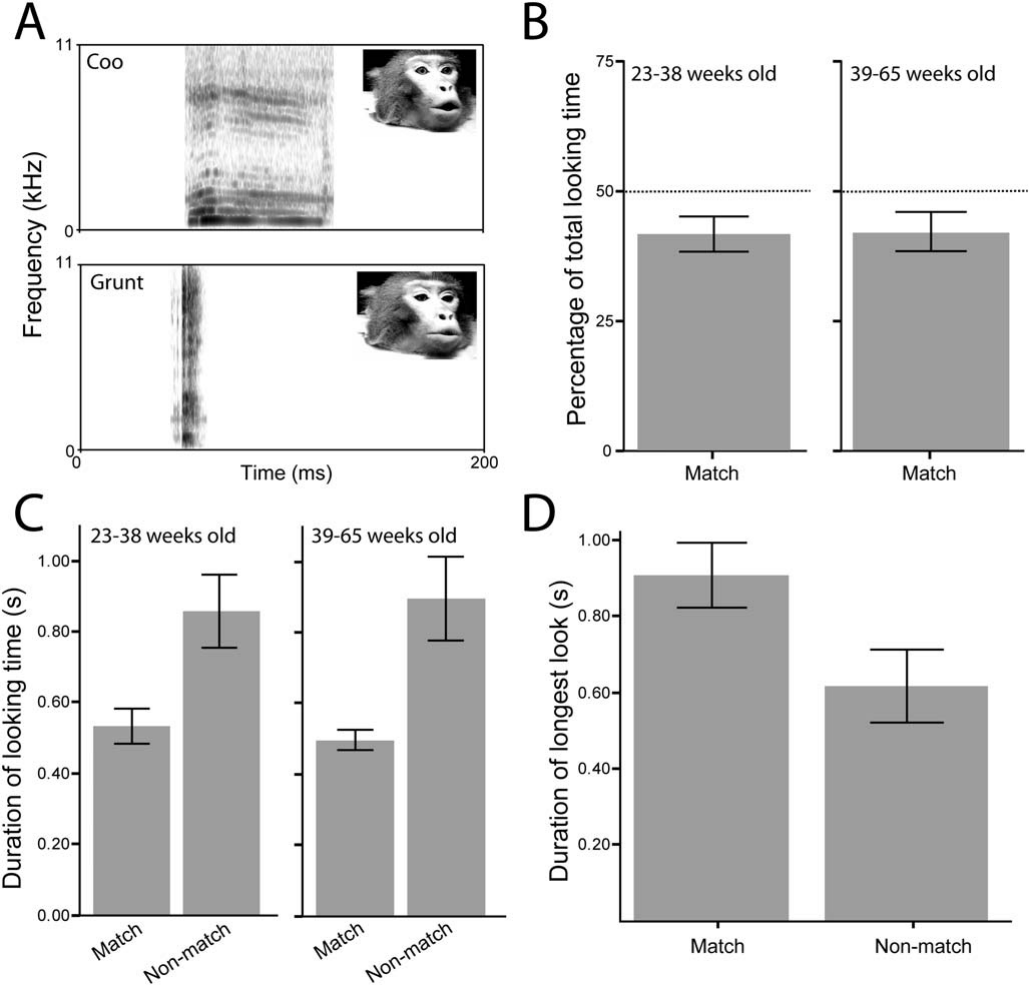
Looking behavior of infant vervets at dynamic audiovisual presentations of macaque calls in Experiment 1. A. Spectrograms of the rhesus monkey coo and grunt. Inset shows one frame of the peak of the corresponding facial expression. B. The percentage of total looking time that the subjects spent looking at the matching face for two age groups. C. The mean duration of looking time at the matched and mismatched faces for the two age groups. D. The mean duration of the longest single look at the matched and mismatched faces. Error bars represent ±1 SEM.

The purpose of our study was to determine whether infant vervet monkeys can recognize the correspondence between the faces and vocalizations of a primate species with which they had no prior experience. To do so, in the first experiment, we compared the amount of looking that vervets accorded to each of two rhesus monkey facial expressions made when vocalizing two different calls—a coo or a grunt—in the presence of the audio version of one of these calls versus looking at the same faces in silence (Figure 1A; please see the Experimental Procedures section for more details). Three patterns of looking were possible. First, the subjects could have spent equal amounts of time looking at both faces. This would have indicated that they did not detect any correspondences across modalities [25]. Second, they could have spent a greater proportion of time looking at the matching face. This is the typical result that is obtained in human infant studies and adult monkey studies [6], [7], [10], [11], [20]. Finally, subjects could have spent a greater proportion of time looking at the non-matching face. Although an atypical outcome, this would still indicate that they recognized the correspondence between the faces and voices and that they systematically *avoided* looking at the matching face. The last two possible outcomes are both meaningful. In essence, the preferential looking method allows us to draw two types of inferences: that audiovisual correspondence has been recognized and that the direction of preferential looking reflects the salience of the stimuli, where salience can be determined by affective and physical properties of the stimuli [26].

We tested infant vervets from 23 to 65 weeks of age (n = 56). To make sure that our results were not affected by the specific monkey presented during test, the particular vocalization presented, and presentation side of particular facial expression, we analyzed the looking time data by way of a repeated-measures analysis of variance (ANOVA) with Caller (i.e., identity of the macaque monkey), Call (i.e., coo vs. grunt) and Side (i.e., the left or the right video screen) as the within-subjects variables. This analysis yielded neither any significant interactions nor any main effects. Then, to determine if there was a perceptual narrowing effect within this age range, we split the subjects into two age groups. The younger group ranged in age from 23 to 38 weeks (mean age = 33 weeks, or ∼8 months) and the older group ranged in age from 39–65 weeks (mean age = 46 weeks, or ∼12 months). The subjects in both age groups spent a greater proportion of their total looking time looking at the non-matching face than at the matching face (23–38 week-olds: paired t-test, t(26) = −2.45, p = 0.021; 38–65 week-olds: t(28) = −2.17, p = 0.039; Figure 1B). In addition, both age groups spent more overall time looking at the nonmatching than at the matching face (23–38 wks old: t(26) = −2.80, p = 0.01; 39–65 wks old: t(28) = −3.45, p = 0.002; Figure 1C).

These patterns of preferential looking are surprising for two reasons. First, both age groups recognized the correspondence between faces and vocalizations even though they are at or beyond the age where perceptual narrowing occurs in human infants (8 months) [24]. Second, the subjects spent more time looking at the non-matching screen. Because this finding is not typical, we computed another index of intersensory matching: the single longest look. Other studies [6], [27], [28] have found that the longest look also provides a useful measure of intersensory matching. For this analysis, we pooled the data from the two age groups because there were no differences in looking patterns between the two age groups and no correlation between age and looking time (r = 0.128, p = 0.372). Results of this analysis indicated that subjects' longest single looks were, on average, directed at the matching than at the nonmatching face (0.91±0.084 vs 0.62±0.096 seconds, t(54) = 2.53, p = 0.014; Figure 1D).

Overall, our findings suggest that vervet infants recognize the correspondence between the faces and vocalizations of another species and that they do so at ages during which human infants already exhibit evidence of perceptual narrowing. At the same time, however, our findings beg the question of why the vervets spent more time looking at the nonmatching face. The answer to this question may lie in two facts. First, in both humans and monkeys, there is a co-modulation of mouth movements with the amplitude envelope of the voice signal [29], [30], [31]. Second, when auditory and visual information is temporally coincident and co-modulated this usually leads to enhanced responsiveness at the neural and behavioral levels [13], [32], [33], [34], [35], [36]. These two facts suggest that the temporal coincidence and co-modulation of the matching face and vocalization was more salient. As a result, we hypothesized that the greater salience of the matching face induced anxiety and fear in our infant vervets and that to reduce the anxiety-provoking nature of this situation [26], our vervets turned away from the nonmatching face. We tested this hypothesis in three different ways.

**Figure 2:**
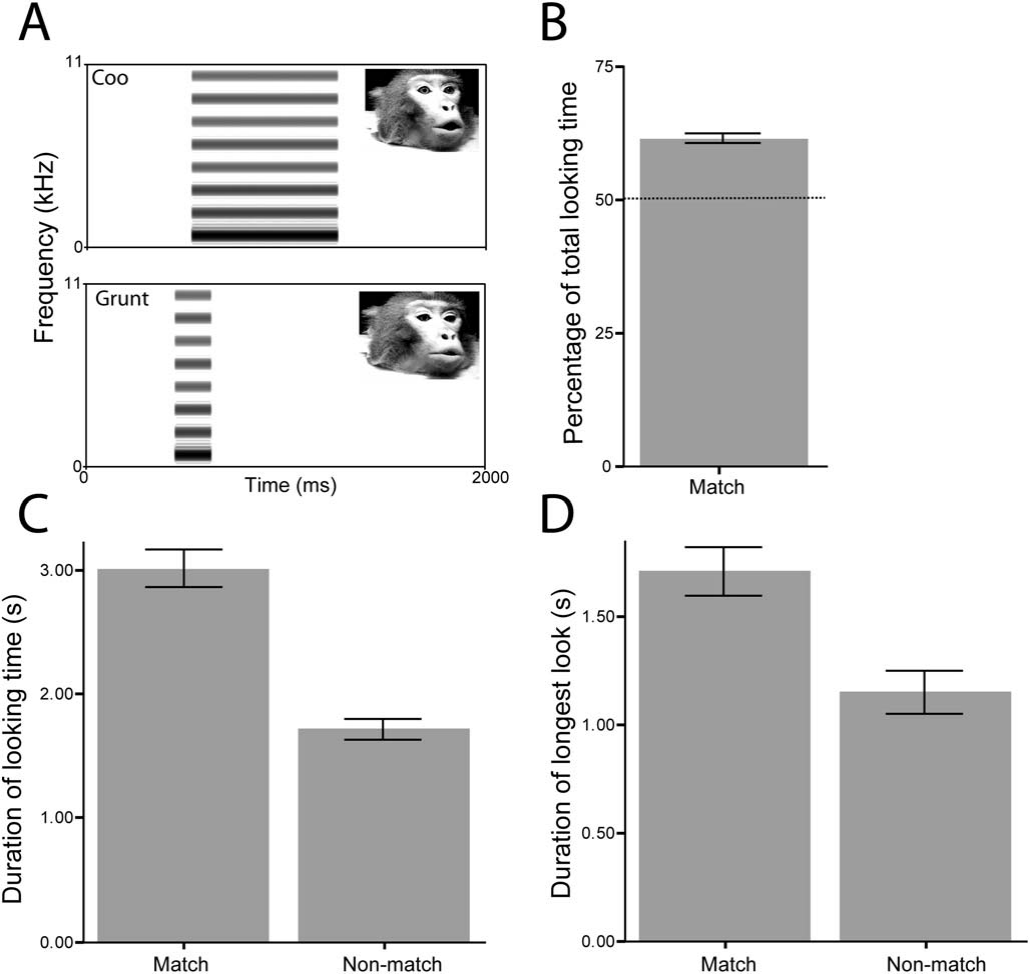
Looking behavior of infant vervets at dynamic audiovisual presentations of rhesus monkey faces paired with complex tones in Experiment 2. A. Spectrograms of the tone stimuli showing that they were matched in duration to the original vocal sound track. The average fundamental frequency of the two vocalizations was used as the fundamental frequency for both complex tones. Inset shows one frame of the peak of the corresponding facial expression. B. The proportion of total looking time that the subjects spent looking at the matched face. C. The mean duration of looking time to the matched and mismatched faces. D. The mean duration of the longest single look at the matched and mismatched faces. Error bars represent ±1 SEM.

First, we tested another group of infant vervets (21–50 wks old) with the same visual stimuli, but with the vocalizations replaced with a complex tone that was broadband and that had the same duration and same average fundamental frequency as the original vocalizations. Importantly, the tone had a constant intensity and a linear spectral profile, and thus lacked the species-specific amplitude envelope and formants that are typically very salient features of speech and nonhuman primate vocalizations [37], [38], [39], [40]. The results were consistent with our predictions. When we degraded the fine-grained spectrotemporal correlations (e.g., amplitude fluctuations) that the voice component bore with respect to the dynamic faces and, thus, reduced the overall salience of the stimulation, the vervet infants still exhibited evidence of intersensory matching. Here, the evidence was in the opposite direction and even stronger than in the first experiment in that subjects looked significantly longer both in terms of percentage of total looking time (Figure 2B; one sample t-test, t(54) = 10.49, p<0.001) and mean duration of looking to the matching (3.01±0.15 s) than to the non-matching (1.72±0.09 s) face (Figure 2C; t(54) = 11.22, p<0.001). Similarly, the longest single looks were to the matching face (Figure 2D; 1.71±0.11 s vs. 1.15±0.01 s; t(54) = 4.32, p<0.001). Together, these data represent one line of evidence that supports our hypothesis that the veridical Face+Voice combination (Figure 1A) is anxiety-inducing and results in longer overall looking at the mismatching face and reduces overall looking. Consistent with this interpretation is the finding that the mean duration of looking at the matching face was 0.8 seconds in the Face+Voice experiment, but that it was more than three times greater in the Face+Complex Tone experiment (3 seconds). As before, a repeated-measures ANOVA revealed no side, sound or face biases. Furthermore, there was no significant correlation between age and looking time (r = 0.018, p = 0.899).

Our second test of the hypothesis that the salience of matching rhesus monkey faces and vocalizations induced anxiety was to measure the relative amount of pupil dilation, both within conditions (match versus nonmatch) and across conditions (Face+Voice versus Face+Complex Tone). While pupils dilate in the dark and constrict in bright light, their diameter is also modulated by the valence of emotional stimuli or their ‘interest’ value [41], [42]. The most reliable pupillary response is dilation towards unpleasant stimuli [41]. Thus, a strong prediction of our hypothesis is that vervets should have a greater pupillary response (in the form of dilation) while viewing the matching rhesus monkey face in the Face+Voice condition than when viewing the nonmatching face but that they should not exhibit this difference in the Face+Complex Tone condition.

**Figure 3:**
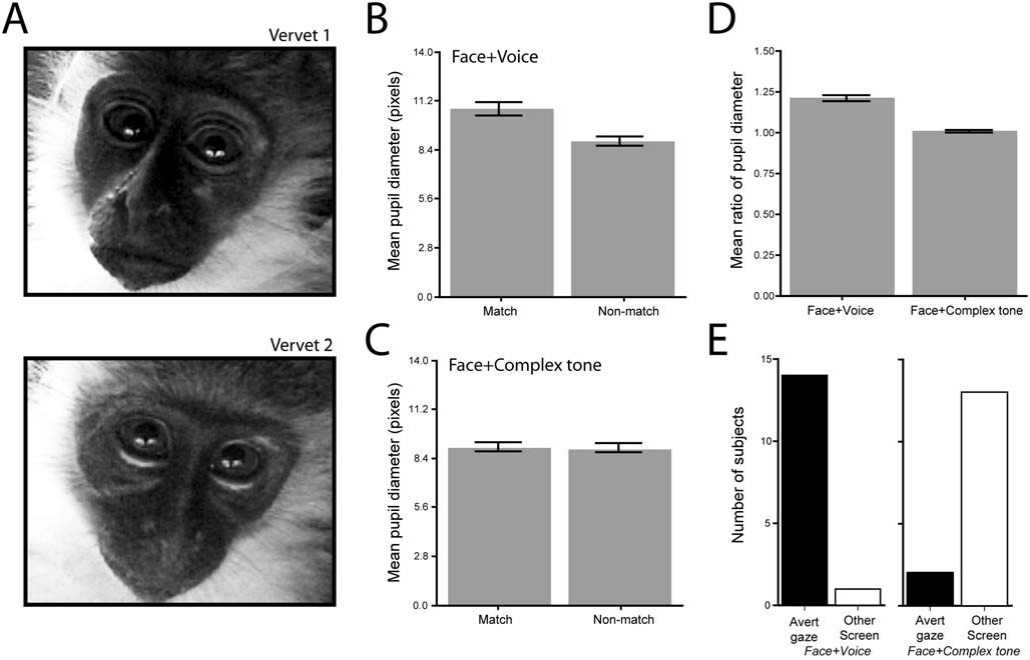
Pupillary response measures to each of the stimulus conditions. A. Two frames from different vervet monkeys showing their dilated pupils in response to viewing the matching face in the Face+Voice condition. B. Mean pupil diameter (in pixels) at the end of the longest single look at the matched face versus mismatched face in the Face+Voice condition. C. Mean pupil diameter (in pixels) at the end of the longest single look at the matched face versus mismatched face in the Face+Complex Tone condition. D. The mean ratio of pupil dilation between matched versus mismatched looks across the two conditions. E. The proportion of subjects that averted their gaze versus those who looked at the other face following their single longest looks in the Face+Voice and Face+Complex Tone conditions. Error bars represent ±1 SEM.

We randomly selected 15 subjects from the Face+Voice and Face+Complex Tone conditions. For each subject, pupil diameter was measured at the end of the ‘longest look’ towards the matching face and at the end of the longest look towards the nonmatching face (Figures 1D and 2D). Figure 3A shows two vervets with their pupils dilated when looking at the matching face. Across our sample, the mean pupil diameter was significantly greater when subjects were viewing the matching versus the nonmatching face (10.82±0.392 vs. 8.988±0.252 pixels; t(14) = 9.62, p<0.0001; Figure 3B). In contrast, in the Face+Complex Tone experiment, no such pupillary response differences were evident (9.19±0.28 vs. 9.14±0.252 pixels, t(14) = 1.09, p = 0.294; Figure 3C). Comparing the match/nonmatch ratio between the two conditions also revealed that pupil dilation was significantly greater in the Face+Voice than in the Face+Complex Tone experiment (1.21±0.018 vs 1.01±0.006; t(28) = 10.65, p<0.0001; Figure 3D). These pupillary response data support our hypothesis in two ways. First, they show that vervet monkeys found the matching face to be more salient than the non-matching face in the presence of the natural vocalization. Second, they show that vervet monkeys found the matching face in the presence of the natural vocalization to be more salient than the matching face in the presence of the complex tone. Importantly, none of the differences in pupillary response can be attributed to differenes in luminance because the videos of rhesus monkeys were recorded under identical conditions, the two conditions used the identical face stimuli, and the matching face was left-right counterbalanced.

To futher test our anxiety-induction hypothesis, we tested the salience of the matching Face+Voice stimuli using a gaze-aversion measure. This measure provides another useful index of whether the vervets found the matching Face+Voice stimuli more arousing than the Face+Complex Tone stimuli. Using the same 15 subjects per condition as in the pupillary response analysis, we scored whether at the end of their longest single looks directed at the matching Face+Voice versus the matching Face+Complex Tone stimuli, the vervets' first response was to avert their gaze from both faces (by looking away or closing their eyes) or to simply look at the other, nonmatching face. Figure 3E shows that for the Face+Voice condition, 14 out of 15 vervets averted their gaze (binomial test, p = 0.0001), while in the Face+Complex Tone condition, only 2 out of 15 vervets averted their gaze; the rest of the subjects looked toward the other face (binomial test, p = 0.007). These data, along with the pupil dilation data, support the hypothesis that the matching Face+Voice stimuli were particularly salient and probably induced anxiety.

## Discussion

By the age of 8 months, human infants no longer match the faces and vocalizations of another species, the rhesus monkey [24], but continue to recognize the intersensory invariance of the faces and vocalizations of their own species. To investigate the evolutionary origins of this developmental process, we tested young vervet monkeys' intersensory response to rhesus monkey faces and vocalizations using similar testing methods and stimulus materials used previously with human infants [24]. We found that vervet monkeys exhibited evidence of cross-species intersensory matching despite the fact that they had no prior experience with macaque monkeys and, thus, that they did not exhibit evidence of perceptual narrowing. The absence of perceptual narrowing in vervets may be due to the precocial neurological development of this species. A comparison of the rate of neural development in vervet monkeys relative to humans indicates that vervets develop at a rate that is 3 to 4 times faster than humans [15], [16]. Therefore, neurologically speaking, our vervet subjects—whose age range was from 23 to 65 weeks (∼6 to 16 months) and mean age was 40 weeks (or 10 months)—were the equivalent of ∼1 to 5 year old human children. This, in turn, means that from a neuro-developmental perspective, the vervets were well beyond the point when perceptual narrowing occurs in humans [20], [21], [24].

How might infant vervet monkeys make cross-species intersensory matches? One likely possibility is that they were simply using temporal cues to recognize the correspondence between the coo face and vocalization and the grunt face and vocalization (albeit, demonstrating the recognition of this correspondence by looking at the mismatching face). The most likely temporal cues that they could have used were onset/offset synchrony of the corresponding visual and auditory cues as well as their common durations [22]. Indeed, it appears that human adults can use the same cues and, thus, readily recognize the correspondence between these very same rhesus monkey expressions [23]. Perhaps the most interesting aspect of the current findings is that vervet monkeys of the same age range as human infants do not exhibit the same developmental pattern of intersensory responsiveness despite the fact that they were tested with the exact same stimuli and with the exact same experimental procedures as were the human infants. Whereas cross-species intersensory responsiveness does not decline in vervet monkey, it does in human infants.

Although the vervets recognized the correspondence between macaque faces and vocalizations by exhibiting differential looking at one face versus the other, they did so by looking at the nonmatching face. We interpreted this pattern of response as a reflection of the increased salience (and perhaps, anxiety-inducing effects) of concurrent face-voice inputs that are temporally co-modulated. That is, we hypothesized that the link between facial movements and vocalizations' amplitude modulations [29], [30], [31] created a particularly salient audio-visual combination and that this induced anxiety. In support of this hypothesis, a second condition revealed that, when the same faces were paired with complex tones that matched the natural vocalization in terms of duration and average fundamental frequency but lacked the species-typical spectral and amplitude envelopes, the vervets now looked longer at the matching face. This finding shows that the co-modulation of auditory and visual signals in the Face+Voice condition determined whether the vervets looked at the matching or non-matching face. Overall, the fact that the vervets exhibited systematic preferences in each experiment indicates that they were linking the visual and auditory information and, thus, perceiving the faces and voices as unitary events.

Although the opposite patterns of looking across the two experiments suggested the anxiety hypothesis, these data do not provide independent evidence that anxiety mediated responsiveness. The pupillary response data do, however, provide such evidence. These data indicated that vervets' pupils dilated significantly more when they looked at the matching face in the Face+Voice experiment but not in the Face+Complex Tone experiment. Although pupil dilation in response to emotionally-arousing stimuli is well-established in humans [42], [43], ours is the first demonstration of similar pupillary responses in monkeys. Consistent with the “anxiety” interpretation, the vervets' overall looking time in the Face+Complex Tone experiment was 3-times higher than in the Face+Voice experiment. Furthermore, they tended to avert their gaze away from both faces after looking at the matching face and listening to the natural voice, but did not do so when looking at the matching face when listening to the complex tone.

One of the interesting questions that our findings of differential patterns of looking to the nonmatching versus matching face raise is why did the vervets in our study behave differently than do rhesus monkeys presented with the same stimuli [6]? First, our vervets were infants whereas the rhesus monkeys were adults. Second, the vervets were viewing and hearing unfamiliar faces and voices; they never had exposure to any heterospecific primates beyond the human caregivers and other staff members at the primate facility. In contrast, the adult rhesus monkeys were viewing and hearing highly familiar conspecific communication signals. Thus, for rhesus monkeys, the familiarity of the communication signals may have attenuated their emotional response to these signals.

Why do infant vervets continue to match hetero-specific faces and voices at a postnatal and neurological age that, based on a comparison with human infants, is beyond the time when intersensory perceptual narrowing should have occurred? There are two possible explanations. One possibility is that monkeys are actually ‘stuck’ with a broader range of sensitivity because of the more precocial nature of their nervous system and, as a result, can integrate the multisensory social signals of their own species as well as those of other related species. The other possibility is that monkeys' precocial brains are not stuck *per se*, but rather are less plastic in the same sense that magnitude of sensory cortical plasticity in older animals is not as great as it is in younger animals [44]. According to this scenario, vervets may still be sensitive to social experience, but it may take them longer to incorporate the effects of such experience and as a result, they may need considerably more postnatal experience to exhibit perceptual narrowing. The latter possibility is consistent with the development of vocal behavior in vervets in that their ability to produce vocalizations, their ability to use them in appropriate contexts, and their responses to the vocalization of conspecifics (and even sympatric heterospecific alarm calls) all emerge gradually during the first four years of life [45], [46]. For example, infant vervets produce ‘eagle’ alarm calls to a very broad class of visual stimuli found in the air above (both harmful and harmless bird species, falling leaves, etc.). Over time, however, they limit their alarm calls to a very limited set of genuinely dangerous raptor species [47]. This suggests that learning of social signals may take a relatively long time in vervets when compared to humans.

That so much postnatal experience (∼4 years) is required for vocal recognition of both conspecific [46] and heterospecific [45] alarm calls suggests that the same may be true for the development of intersensory perception of conspecific signals. Ideally, to distinguish between the two possibilities offered above–no perceptual narrowing versus slow perceptual narrowing–and under natural conditions, it would be necessary to test adult vervets using the same procedures and stimuli as used in the current study. This remains a future direction of the current work. At a minimum, such future work will require substantial modification of the experimental procedures to accommodate adult subjects. Nonetheless, there is suggestive evidence that adult capuchin monkeys can match the faces and vocalizations of other primate species with which they have had no prior experience [48]. Given that the capuchins have a similar precocial time course for brain growth [14], this suggests that adult vervets are likely to exhibit cross-species intersensory matching into adulthood and, thus, that vervets' intersensory perceptual responding to other monkey species never declines.

Although the possibility that either no perceptual narrowing occurs or that perceptual narrowing occurs slowly in monkeys is reasonable given the existing data, there is an alternative explanation that is possible as well. Our vervets were captive and, therefore, exposed to a broader than normal array of faces and voices (conspecifics and humans). As a result, it is possible that our vervets' species-atypical experience with humans either may have broadened their intersensory perceptual capacities or extended the sensitive period for perceptual narrowing. This interpretation is supported by two lines of evidence. First, Japanese macaques reared for many months with no exposure to faces have a broad perceptual sensitivity to both human and monkey faces[49]. When these face-deprived monkeys were subsequently exposed to monkey or human faces exclusively for one month, they then showed a perceptual narrowing bias towards only the exposed species' faces. These data underscore the importance of experience in driving perceptual narrowing and suggest that the “window” of the sensitive period when face sensitivity is initially broad may be determined by the timing of exposure. It also raises the possibility that, had they been exposed to the faces of both species, they would have shown broader face recognition abilities. Second, studies of the sensitive-period of song learning by birds also reveal the importance of exposure and experience, but in the opposite direction. In zebra finches, the sensitive period for song-learning can be extended by manipulating the social contexts in which the ‘tutor’ song is heard [50], even for songs of another species [51]. A similar process may allow infant vervets to recognize the face/voice correspondences in species they are not familiar with.

The current findings provide important new information and insights regarding perceptual and intersensory development in vervet monkeys. First, they show that infant vervets can integrate unfamiliar auditory and visual information suggesting that intersensory integration mechanisms are robust and highly conserved in evolution. Second, they show that the specific patterns of intersensory integration depend on whether the context is a social and ecologically meaningful one or an abstract one. Our data provide the first evidence of cross-species intersensory matching in a developing non-human primate species. Future studies will need to determine whether, when, and how perceptual experience shapes the ultimate organization of perceptual systems in non-human primates and, in particular, the developmental trajectory of intersensory integration mechanisms. Data such as these will, in turn, inform cross-species comparisons and, thus, will suggest ways in which the evolution and development of intersensory integration mechanisms are related.

## Materials and Methods

All experimental protocols and procedures were approved by the Animal Care Committee of McGill University and the Montreal Neurological Institute and Hospital Research Ethics Board.

### Subjects

The subjects consisted of two groups of infant vervet monkeys (*Cercopithecus aethiops*). In the first experiment, the group consisted of 56 animals with an age range of 23–65 weeks and a mean age of 37 weeks. In the second experiment, the group, chosen from a separate cohort of vervets, consisted of 55 animals ranging in age between 21 and 50 weeks with a mean age of 31 weeks. Both groups were randomly selected from the offspring of a larger colony of adult vervets maintained at the Behavioural Sciences Foundation Laboratories located in St Kitts, West Indies. The subjects were naïve to our experimental procedures, and they had not been used for any experiments previously. In addition, these monkeys had never been exposed to other species of primates, except for humans.

### Apparatus

An animal technician, blind to the purposes of our experiments, held a subject while seated in the centre of a three-sided enclosure whose sides were covered by a thick dark curtain to isolate the subject from the experimenter. In the middle of this enclosure, a 20-inch Apple Cinema Display presented the video component of the audio-visual stimuli. This LCD panel was connected to an Apple MacBook Pro with a 2.0 GHz Intel Core Duo processor, which was used to deliver the stimuli on a trial-by-trial basis. The audio component of the stimuli was presented using a single speaker connected to the laptop computer. The speaker was placed in the middle and under the LCD panel. A Canon Optura 600 digital video camcorder was mounted on a Manfrotto video tripod and placed in the middle and above the LCD panel in order to provide a live feed of all experiment sessions to the experimenter and to record the looking behavior of each subject for subsequent off-line analysis.

### Stimuli

We tested the vervets with three different stimulus sets. In Experiment 1 with the first group of vervets, we tested them with the coo and grunt calls of the rhesus monkey (*Macaca mulatta*). Two sets of calls were used and produced by two different rhesus monkeys. Figure 1A shows exemplars of one of the two call pairs we used in this experiment. In addition, Videos S1 and S2 provide samples of the actual face-voice stimuli used to test our subjects. The duration of each video clip containing each call was 2 seconds and the videos were temporally aligned to the onset of mouth movements. It is important to note here that rhesus monkeys look very different from vervet monkeys. The former have a pinkish face surrounded by tan-colored fur, while the latter have black faces fringed with white fur and then surrounded by a greenish brown fur. In addition, vervet monkeys do not produce coo calls; nearly all Old World monkeys produce a grunt-like call. The coo call was on average 715.3 msec long while the average duration of the grunt call was 142.3 msec based on two macaque callers.

It should also be noted that there is a natural delay for every vocalization (including human speech) between the onset of mouth movements and the onset of the voice. This delay can vary considerably (from tens to a few hundred milliseconds) across both different call types and across different exemplars of the same call type. The pattern of reported results reveals that this temporal factor had no impact on intersensory matching. The same is true for previous results in human infants [22], [24] and adult rhesus monkeys [6]. For a description of such face-voice delays in rhesus monkey vocalizations, see [34], [52].

In the second experiment with the second group of vervets, we used two different stimulus sets. In one stimulus set, the vocal component of the stimuli used in Experiment 1 was replaced with a complex tone (triangular waveform, Adobe Audition 1.5) that matched the call's duration but removed any temporal modulation in the envelope of the signal. The fundamental frequency (F0) of the complex tone was based on an average between the fundamental frequencies of the coo and the grunt of one individual.

For both stimulus sets, the pairs of videos were presented side-by-side in the center of the LCD panel on a black background such that each video frame measured 15.7 cm wide by 10.4 cm high with a horizontal distance of 11.4 cm between the closest edges of the two frames. When a subject looked at the fixation cross located at the center of the LCD panel at a viewing distance of approximately 40 cm from the LCD panel, each video frame subtended approximately 20° of visual angle on either side of the fovea. The audio track was played at approximately 73 dB sound pressure level measured at the subject's ears.

### Experimental procedures

All procedures described here were approved by the appropriate research ethics board at both Montreal Neurological Institute and McGill University and animals were treated in accordance with the guidelines laid out by the Declaration of Helsinki. We used an intersensory paired-preference procedure to determine whether subjects could match the audio signal to the corresponding visual stimulus. A correct match was judged to have occurred if the subject looked longer at the corresponding than the non-corresponding visual stimulus in the presence of the sound than in its absence. In Experiment 1 (i.e., Face+Voice condition), the looking behavior of each subject was recorded during eight 20-sec trials. A single trial consisted of a 4-sec silent presentation of two calls made by the same macaque monkey followed by 16 seconds during which the two videos were presented together with a single audio track matching the content and duration of only one of the two videos. The total of 8 trials ensured that each subject was exposed to all arrangements (i.e., left vs. right), callers (i.e., two macaques), and call types (i.e., coo vs. grunt) for which a match between a video and the audio streams could be made.

In Experiment 2 (i.e., Face+Complex Tone condition), with two other stimulus sets, the procedure was virtually identical to that of Experiment 1 except that looking behavior was recorded during twelve 20-sec trials, clustered into four 3-trial groups. Each subset of 3 trials began with a silent trial during which two side-by-side videos of the same macaque monkey mouthing two different calls or two side-by-side different-duration checkerboards were presented repeatedly. This was followed by two trials (counterbalanced for side of presentation) during which subjects saw the same two videos and a single audio track that matched the onset/offset of only one of the two videos.

A coder, who was blind to the testing conditions and to the stimuli being presented on a given trial, measured the direction of looking (i.e., left video frame, right video frame or away from the monitor) and the duration of each look throughout each trial. Inter-observer reliability was computed on a sample of randomly chosen subjects. The average level of agreement on the total duration of looking on each side per trial was 96% for Experiment 1 and 97% for Experiment 2.

### Pupillary response measures

Fifteen subjects were randomly selected from each of the Face+Voice and Face+Complex Tone conditions. For each subject two measurements of pupil diameter were obtained: one at the end of the longest look at a matching video and the other at the end of longest look at the non-matching video. Pupil diameter measurements (in pixels) were made by first enhancing the contrast of each image in Adobe Photoshop CS3. Then a circular marquee was placed at the boundary that separated the black pupil region from the dark amber color of the iris of the same eye in each captured frame. A ratio of pupil diameter was computed by dividing the measurement in the matching by the nonmatching pupil diameters. In addition, we scored whether, following the single longest look, subjects looked away from both video frames (averted gaze) or looked toward the other video frame.

## Supporting Information

Video S1 Coo-Grunt pair of the first macaque presenter. The matching video is on the left side.
(1.88 MB MOV)

Video S2 Coo-Grunt pair of the second macaque presenter. The matching video is on the right side.
(1.55 MB MOV)
